# Cognitive trajectories: exploring the predictive role of subjective cognitive decline and awareness of age-related changes for cognitive functioning

**DOI:** 10.3389/fpsyt.2023.1270798

**Published:** 2023-10-19

**Authors:** Serena Sabatini, Stephanie Cosentino, Silvia Chapman, Clive Ballard, Helen Brooker, Anne Corbett, Blossom C. M. Stephan

**Affiliations:** ^1^School of Medicine, Institute of Mental Health, University of Nottingham, Nottingham, United Kingdom; ^2^Taub Institute for Research on Alzheimer’s Disease and the Aging Brain, New York, NY, United States; ^3^Department of Neurology, Columbia University, New York, NY, United States; ^4^Faculty of Health and Life Science, University of Exeter, Exeter, United Kingdom; ^5^Ecog Pro Ltd., Bristol, United Kingdom; ^6^Faculty of Health Sciences, Curtin enAble Institute, Curtin University, Bentley, WA, Australia

**Keywords:** AARC-gains, AARC-losses, self-perceptions of aging, subjective cognitive decline, objective cognitive change

## Abstract

**Background:**

We investigated whether aspects of subjective cognitive aging, including awareness of age-related gains and losses in cognition (AARC-gains, AARC-losses) and subjective cognitive decline (SCD), predict change in objective cognitive function as measured by verbal reasoning (VR) and working memory (WM).

**Methods:**

We used longitudinal data for 3,299 cognitively healthy UK residents aged 65+. We used data on AARC and SCD assessed in 2019, and cognitive tasks assessed in 2019, 2020, and 2021. We used latent growth curve modeling, latent class growth analysis, and growth mixture modeling.

**Results:**

For VR, multiple growth trajectories were not evident. Mean VR at baseline was 37.45; this remained stable over time. Higher AARC-gains in cognition (mean intercept = −0.23; 95%CI: −0.31; −0.16), higher AARC-losses in cognition (mean intercept = −0.37; 95%CI: −0.46; −0.28), and lower SCD (mean intercept = 2.92; 95%CI: 2.58; 3.58) were associated with poorer VR at baseline. A three-class growth mixture model–class varying best represented trajectories of WM. In Class 1 (*N* = 182) mean WM at baseline was 31.20; this decreased by 2.48 points each year. In Class 2 (*N* = 119) mean WM at baseline was 23.12; this increased by 3.28 points each year. In Class 3 (*N* = 2,998) mean WM at baseline was 30.11; and it remained stable. Higher AARC-gains (Odds Ratio = 1.08; 95%CI: 1.03; 1.14) and AARC-losses (Odds Ratio = 1.10; 95%CI: 1.04; 1.16) in cognition predicted greater likelihood of being in Class 2 than Class 3.

**Conclusion:**

Although both higher AARC-gains and AARC-losses indicate poorer concurrent cognition, higher AARC-gains may be a resource that facilitates future cognitive improvement.

## Introduction

The global number of older people is rapidly increasing, and with that also the number of people experiencing some level of cognitive decline (1). The majority of older individuals experience normal levels of decline (i.e., age-related cognitive decline) in cognitive domains such as processing speed, reasoning, memory, and executive function (2). Still, a relevant proportion of older individuals experiences pathological levels of cognitive decline (i.e., mild cognitive impairment and dementia) (3). Indeed, the global proportion of people aged 60+ having mild cognitive impairment ranges between 12 and 18% and the European proportion of people aged 60+ having dementia is about 6.5% (4–6). Due to the high economic and social cost of pathological cognitive decline, promoting maintenance of cognitive functioning is a global priority (7). Identification of individuals at higher risk of future cognitive decline provides an opportunity to implement interventions aiming to prevent or delay pathological cognitive decline.

### Self-perceptions of aging as predictors of change in cognitive function

A psychological construct that may help to detect individuals at higher risk of cognitive decline is Self-Perceptions of Aging (SPA). SPA capture individuals’ perceptions and evaluations of the changes they experience while growing older, including changes in cognitive domains. Generally, those individuals with more positive SPA show better objectively-assessed cognitive functioning in cross-sectional studies (8), and have less risk of future cognitive decline (9) and of receiving a diagnosis of dementia (10). More positive SPA are also related to lower risk of developing Alzheimer’s disease biomarkers including declining hippocampal volume, and accumulation of amyloid plaques and neurofibrillary tangles (11). It has even been found that positive SPA attenuate the likelihood of developing dementia among at risk individuals such as APOEε4 carriers (12). However, some studies find no significant association between SPA and cognitive functioning (13, 14). Mixed findings may be due to differences across studies in the measures of SPA used (e.g., unidimensional versus multidimensional), cognitive abilities examined (e.g., fluid versus crystallized cognition), and statistical methods (i.e., covariates included in analytical models).

To date, many studies linking SPA and objectively-assessed cognitive functioning have relied on unidirectional measures (15, 16) that characterize individuals as having either positive or negative SPA. However, positive and negative changes typically co-exist throughout the adult lifespan (17). For example, in the cognitive domain, older individuals often experience an increase in knowledge but a decline in their memory (2). As negative perceptions of aging pervade western societies (18), it is important to obtain a better understanding of the relationship between SPA and cognitive functioning using measures that assess both positive and negative SPA.

### Subjective cognitive decline and awareness of age-related change

Several constructs in the literature can be conceptualized as falling under the umbrella of SPA, including Subjective Cognitive Decline (SCD) and Awareness of Age-Related Changes (AARC). SCD, the self-experienced worsening of cognitive functioning in the absence of objectively measured cognitive impairment (19), is increasingly considered to be a risk state for Alzheimer’s disease. More severe SCD often predicts future cognitive decline (20, 21) and dementia (22), but not always (23). Indeed, SCD has been operationalized in myriad ways (24), with certain measurement factors optimizing its association with AD biomarkers and tasks sensitive to very early Alzheimer’s disease (AD) (25–27).

A concept related to, but distinct from SCD, is Awareness of Age-Related Changes (AARC) (28–30). Whereas SCD is generally conceptualized as cognitive decline that individuals attribute to a pathology (19), AARC-cognition is intended to capture any cognitive change that individuals attribute to their increased age. A 50-item AARC questionnaire (31, 32) makes it possible to separately assess the gains and losses individuals may notice in five life domains and which they attribute to aging including cognition, socio-emotional functioning, physical health, interpersonal relations, and lifestyle. The cognitive subscales from the AARC 50-item questionnaire (hereafter referred to as AARC-gains in cognition and AARC-losses in cognition, respectively) assess perceived losses in areas including processing speed, memory, and mental capacity and perceived positive changes in areas including knowledge, wisdom, and reflexivity. Whereas the multidimensionality of the AARC questionnaire makes it possible to separately assess and link perceived positive and negative changes in cognition and link such changes to objective cognitive functioning, most measures of SCD [e.g., IQCODE; (33)] provide only an overall score indicating either perceived cognitive decline or perceived cognitive improvement. So far it has never been explored whether this scoring difference leads to different associations between AARC and SCD with objective cognitive change. Finally, whereas some SPA concepts, such as attitudes toward one’s own aging (34) are mostly unconscious, AARC is theorized as a component of self-knowledge.

Cross-sectional studies have linked AARC to SCD and poorer objective cognitive function (35). Sabatini et al. (32) found that, among UK individuals aged 50+, higher AARC-losses in cognitive functioning showed a moderate association with greater SCD. Sabatini et al. (36) found that, among UK individuals aged 50+, those perceiving more cognitive losses had poorer objectively-assessed cognition; especially poorer memory and grammatical reasoning. Testad et al. (37) found that, among Norwegian individuals aged 50+, higher AARC-losses across life domains correlated with poorer scores on computerized cognitive tasks assessing visual, spatial, and numeric working memory, grammatical reasoning, and executive functions. Voelkner and Caskie (38) found that, among US adults aged 66–90 years, higher AARC-losses across life domains were related to poorer scores on tasks assessing memory and inductive reasoning. Finally, analyses of daily diary data from US individuals aged 60–90 years showed that AARC-losses across life domains predicted within-person decreases in objectively-assessed inductive reasoning on the same day and decreases from 1 day to the next (39).

So far longitudinal evidence linking AARC to objectively-assessed cognitive functioning has relied solely on global scores for AARC-gains and AARC-losses across life domains rather than in cognition specifically. Sabatini et al. (40), found that greater 20-year decline in processing speed predicted higher AARC-losses across life domains in individuals aged 60+ at baseline. This suggests that individuals’ perceptions of age-related losses may be useful to capture a past trajectory of cognitive decline. Finally, a recent study, that explored the one-year bidirectional influence of AARC across life domains with cognitive functioning found that both AARC-losses and scores on cognitive tasks had small effects on each other (Sabatini et al., under review). Nonetheless, these results suggest that AARC may influence and/or help predict future cognitive change.

With regards to AARC-gains, evidence thus far has produced counterintuitive results. At a cross-sectional level, lower AARC-gains were associated with better performance on spatial working memory and grammatical reasoning among 50+ UK individuals (36). Lower AARC-gains were also associated with better grammatical reasoning among Norwegian individuals aged 50+ (37). Finally, lower AARC-gains were related to better performance on inductive reasoning among US individuals aged 66–90 years (38). Overall, both higher AARC-gains and higher AARC-losses seem to be related to poorer objectively-assessed cognitive functioning. However, longitudinal evidence is currently scarce. There is no longitudinal exploration of matched associations between AARC in cognition and performance on cognitive tasks. This would be important as domain-specific measures of SPA may have additional value in predicting matched outcomes (41).

### The current study

This study aimed to investigate, in a large sample of cognitively healthy UK individuals aged 65+, whether levels of AARC-gains in cognition, AARC-losses in cognition, and SCD at baseline predict verbal reasoning (VR) and working memory (WM) scores over time. We focused on individuals aged 65+ as younger individuals are less likely to experience significant cognitive change over 2 years. We expected higher AARC-losses in cognition and greater SCD to be related to poorer VR and WM at baseline and to greater decline in VR and WM over the study period of 2 years. Given the counterintuitive direction of the previously found associations between higher AARC-gains and poorer cognitive performance, we framed the longitudinal associations of AARC-gains in cognition with VR and WM over time as exploratory.

## Materials and methods

### Study design and protocol

This study used data collected online through the UK PROTECT (Platform for Research Online to investigate Genetics and Cognition in Ageing) study.^1^ To enroll, individuals needed to be UK residents, English speakers, aged 50+, have internet access, lack a clinical diagnosis of dementia at baseline (which started in 2015), and provide informed consent online. During recruitment, the study was publicized nationwide and among cohorts of older adults including *Exeter 10,000*, *Join Dementia Research*, and *Brains for Dementia Research*. UK PROTECT obtained ethical approval from the London Bridge NHS Research Ethics Committee and Health Research Authority (Ref:13/LO/1578).

In UK PROTECT, participants were invited to take part in a yearly follow-up assessment. For the scope of this study, as part of their assessment in January 2019, participants were asked to fill in additional questions assessing AARC. A total of 6,658 individuals provided data on AARC in 2019 and on cognitive performance in at least two of the assessments taking place in 2019, 2020, and 2021. Out of these individuals, 3,094 were excluded from the current study analytical sample as they were younger than 65 years and hence did not meet the current study inclusion criteria.

Additional exclusion criteria were put in place to omit from the analytical sample individuals with possible pathological cognitive decline at baseline including: (1) *n* = 51 individuals as they reported, in the Lawton’s Instrumental Activities of Daily Living questionnaire (34), needing help managing medications and/or managing household finances (suggesting cognitive impairment); (2) *n* = 19 individuals as they self-reported a clinical diagnosis of mild cognitive impairment; and (3) *n* = 195 individuals who obtained a score > 3.3 on the Informant Questionnaire on Cognitive Decline in the Elderly short form-informant version (33); indicating possible pathological cognitive decline (42). Therefore, the resulting analytical sample comprises 3,299 participants.

### Measures

*Awareness of Age-Related Change (AARC) in cognition* was assessed with the cognitive functioning subscale of the AARC-50 questionnaire (31, 32). This subscale includes ten items, five assessing perceived gains in cognition and five assessing perceived losses in cognition. An example of gain item is “*With my increasing age, I realize that I have become wiser*,” whereas an example of loss item is “*With my increasing age, I realize that I am more forgetful*.” Respondents rate how much each item applies to them on a five-point Likert scale (1 = *not at all*, 2 = *a little bit*, 3 = *moderately*, 4 = *quite a bit*, and 5 = *very much*). Scores for AARC-gains in cognition and AARC-losses in cognition are obtained by summing items that fall into the respective subscales. Subscales scores can range from five to 25; higher scores indicate higher levels of perceived cognitive gains and losses. Cronbach’s alphas in this sample were 0.87 and 0.86 for AARC-gains and AARC-losses in cognition, respectively.

*Subjective Cognitive Decline (SCD)* was assessed with the self-reported version of the Informant Questionnaire on Cognitive Decline in the Elderly short form [IQCODE-Informant; (33)]. The IQCODE is a 16-item questionnaire that asks respondents to rate changes in cognitive and functional abilities over the last 10 years. Developed for the screening and evaluation of dementia, the IQCODE items query fairly fundamental cognitive and functional abilities, including remembering things about family and friends, remembering address and phone number, following a story in a book, handling money for shopping, etc. The response scale incorporates both improvement and decline (an example item is “*Making decisions on everyday matters*”) and can be answered on a five-point scale (1 = *much improved*, 2 = *a bit improved*, 3 = *not much change*, 4 = *a bit worse*, and 5 = *much worse*). The final score is the mean of the item scores. Hence participants’ scores lie on a continuum with 5 indicating greatest cognitive decline and 1 indicating greatest cognitive improvement. Cronbach’s alpha in this sample was 0.85.

*Cognitive functioning* was measured with the PROTECT Cognitive Test Battery (43) which comprises four tasks: Verbal Reasoning (VR); Paired Associate Learning (PAL) assessing visual episodic memory; Self-Ordered Search (SOS) assessing spatial working memory; and Digit Span (DS) assessing verbal working memory. For each task, a summary score can be obtained by subtracting the number of errors from the number of correct answers; higher scores indicate better performance on each task. For VR, the summary score is obtained by subtracting the number of errors from the number of correct answers, but the score has no upper or lower limit due to the fact that respondents can make attempts on as many trials as they can within the given time of 3 min. For PAL, the summary score can range from 0 to 16. The summary score for SOS can range from 0 to 20. Finally, for the DS task, the summary score can range from 0 to 20. A composite score for Working Memory (WM) can be obtained by summing participants’ scores on PAL, SOS, and DS. Again, higher scores indicate better WM.

*Socio-demographic variables* comprised ethnicity, age, sex, education (*secondary education; post-secondary education; vocational qualifications; undergraduate degrees; post-graduate degrees; doctorates*) and working status (*working; not working*).

### Study analyses

Descriptive statistics for study variables at baseline were reported, and estimated using STATA. We estimated bivariate associations among AARC-gains, AARC-losses, and SCD at baseline.

We investigated trajectories of performance on VR and WM with two models operationalized in Mplus. First, to determine whether and how performance on VR and WM changed between 2019 and 2021 in study participants, latent growth curve modeling (LGCM) was conducted using three waves of UK PROTECT data (2019, 2020, and 2021). Each LGCM estimated the mean intercept (baseline) and the mean slope (change over time) of the variable of interest, with random effects to account for variation across individuals (44). We first investigated associations of AARC-gains in cognition, AARC-losses in cognition, and subjective cognitive decline. Unadjusted and adjusted (for age, sex, education, and working status) models were estimated. We reported the Comparative Fit Index and Tucker-Lewis Index as indicators of model fit. In both indices, values >0.90 indicate excellent model fit (45). For LGCM, missing data on outcome measures were handled using Full Information Maximum Likelihood (FIML) (46), which allows computation of parameter estimates on the basis of all available data without imputing or dropping data when missing under the assumption that data are missing at random (MAR). Through use of LGCM, we assumed that growth trajectories of all individuals in VR and WM can be adequately described using a single estimate of growth parameters.

In a second step, we employed latent class growth analysis (LCGA) and growth mixture modeling (GMM) to examine whether multiple growth trajectories of VR and WM existed in the sample. Different assumptions were tested. The LCGA fixes variances of the global growth factors to zero across classes, assuming trajectories within a class are homogeneous. The growth mixture model-class invariant (GMM-CI) constrains the variances of the global growth factors across classes to be equal (homogeneous classes), and the growth mixture model class-varying (GMM-CV) freely estimated all variances of the global growth factors across classes. A frequent problem with these models is non-convergence (47), with more complex models, particularly those with free variances, more likely to experience convergence difficulties (48). Between one and four class solutions were tested for each assumption, with 1,000 random starts and 20 iterations for each model to avoid local solutions. Following successful convergence, the optimal number of distinct trajectories were determined using Bayesian Information Criterion (BIC), sample size adjusted BIC (ssBIC), the Lo–Mendell–Rubin likelihood ratio test (LMR-LRT) and the bootstrapped ratio test (BLRT) which provide between model comparison (k vs. k-1), and entropy (49). Entropy is a standardized index of model-based classification accuracy based on the average posterior probability, with higher values indicating clearer class separation (50). Substantive criteria were based on a class size greater than 2% and theoretical and practical interpretability of the classes.

GMMs allow for estimation of predictors of class membership (44). The categorical latent class is related to the covariates by way of multinomial logistic regression which assigns each individual fractionally to all classes using posterior probabilities. Predictors of class membership were examined using the “3-step” approach in Mplus (R3STEP) in order to protect the latent class structure from influences of the covariates (51). The posterior probability of class membership was used to investigate whether AARC-gains in cognition, AARC-losses in cognition, and SCD were associated with each class in a multinomial regression model adjusted for age, sex, education, and working status. Changes in the intercept and slope were considered significant if Odds Ratio 95% confidence intervals did not span 1.

Latent trajectory model selection: Results for VR are displayed in Supplementary Table S1. Results for WM are displayed in Supplementary Table S2. Given all available information including model fit indices, interpretability, and theoretical considerations, the optimal model that converged and produced an admissible solution was the two-class GMM-CI model for VR and the three-class GMM-CV for WM. However, for VR, one class comprised only 1% of the sample so change over time in VR was better represented with LGCM.

## Results

### Descriptive statistics

In total, 3,299 individuals (mean age = 70.89 years) met the inclusion criteria. Descriptive statistics of study variables at baseline are presented in Table 1. Most participants were of white race (98.83%) and almost three quarters were women. Educational achievement varied among participants, ranging from completion of secondary education to obtainment of a doctorate. At baseline, 16.4% of participants were employed. Participants’ mean score on the IQCODE-self suggests that on average they did not perceive much change in their cognitive functioning. On average participants reported “few” AARC-gains and AARC-losses in cognition.

**Table 1:** Descriptive statistics for study variables in 2019 (baseline).

Variables	Values
Ethnicity, n (%)	
White: English/Welsh/Scottish/Northern Irish	3,095 (93.8)
White: Irish	40 (1.2)
White: Gypsy Irish Traveller	1 (0.03)
White: European	96 (2.9)
White: Non-European	28 (0.9)
Mixed: White and Black Caribbean	1 (0.03)
Mixed: White and Asian	7 (0.2)
Any other multiple ethnic background	4 (0.1)
Indian	9 (0.3)
Pakistani	2 (0.1)
Chinese	4 (0.1)
Any other Asian background	4 (0.1)
Arab	1 (0.03)
Any other ethnic background	7 (0.2)
Age; M (SD)	70.89 (4.53)
Sex; n (%)	
Men	647 (26.3)
Women	1813 (73.7)
Education; n (%)	
Secondary education	384 (15.6)
Post-secondary education	282 (11.5)
Vocational qualification	479 (19.5)
Undergraduate degree	805 (32.7)
Post-graduate degree	404 (16.4)
Doctorate	106 (4.3)
Working; n (%)	401 (16.4)
AARC-gains in cognition; M (SD)	13.09 (3.70)
AARC-losses in cognition; M (SD)	10.77 (3.05)
IQCODE-self; M (SD)	3.08 (0.21)
Digit span; M (SD)	7.67 (1.49)
Missing; n	48
Paired associate learning; M (SD)	4.68 (0.92)
Missing; n	51
Self-ordered search; M (SD)	7.77 (2.22)
Missing; n	56
Verbal reasoning; M (SD)	37.45 (10.30)
Missing; n	52

*N* = 3,299.

### Bivariate associations for awareness of positive and negative age-related changes in cognition and subjective cognitive decline with demographic variables at baseline

At baseline, older age was correlated with higher AARC-losses in cognition (*r* = 0.12; *p* < 0.001) and with greater SCD (*r* = 0.06; *p* = 0.015), but was not significantly correlated with AARC-gains in cognition (*r* = 0.00; *p* = 0.999). At baseline, sex was not significantly correlated with AARC-losses in cognition (*r* = −0.01; *p* = 0.465), but being a woman was correlated with higher AARC-gains in cognition (*r* = 0.11; *p* < 0.001) and with less SCD (*r* = −0.08; *p* = 0.001). Greater education was correlated with lower AARC-losses in cognition (*r* = −0.08; *p* = 0.002), lower AARC-gains in cognition (*r* = −0.08; *p* < 0.001), and greater SCD (*r* = 0.07; *p* = 0.002). Finally, being employed was correlated with lower AARC-losses in cognition (*r* = −0.08; *p* < 0.0001) and with less SCD (*r* = −0.06; *p* = 0.009), but was not significantly correlated with AARC-gains in cognition (*r* = 0.03; *p* = 0.131).

### Bivariate associations among awareness of positive and negative age-related changes in cognition and subjective cognitive decline

At baseline AARC-gains in cognition were significantly and positively correlated to AARC-losses in cognition (*r* = 0.10; *p* < 0.001). At baseline both AARC-gains in cognition (*r* = −0.06; *p* < 0.001) and AARC-losses in cognition (*r* = −0.04; *p* = 0.019) were significantly and negatively correlated with scores on the IQCODE-Self. Hence both higher AARC-gains and higher AARC-losses were correlated with greater SCD.

### Subjective cognitive aging concepts as predictors of verbal reasoning over time

As we did not find the presence of multiple growth trajectories for VR in the sample, we instead describe results from LGCM (Table 2). Mean score on VR at baseline was 37.45, and it did not significantly change over time. Higher AARC-gains in cognition (adjusted model mean intercept = −0.23; 95% CI: −0.31; −0.16) and higher AARC-losses in cognition (adjusted model mean intercept = −0.37; 95% CI: −0.46; −0.28) were related to poorer scores on VR at baseline. Both higher AARC-gains in cognition (adjusted model mean slope = −0.06; 95% CI: −0.09; −0.03) and higher AARC-losses in cognition (adjusted model mean slope = −0.05; 95% CI: −0.09; −0.02) were related to a slightly greater decline in VR over time. However, these associations were very small. Higher scores on the IQCODE-Self, indicating greater subjective cognitive decline, were related to better VR at baseline (adjusted model mean intercept = 2.92; 95% CI: 2.58; 3.58), but not to change in VR over the 2 years follow-up.

**Table 2:** Latent growth curve model estimating baseline levels and change over time in verbal reasoning.

Verbal reasoning				
	Intercept mean	Slope mean	CFI	TLI
Unconditional model	67.34 (62.68; 72.00)	0.67 (−1.21; 2.55)	0.997	0.992
Variance	69.28 (64.48; 74.07)	0.49 (−1.53; 2.51)		
Latent growth curve model estimating predictors of verbal reasoning at baseline and over time				
Predictors	Intercept mean	Slope mean	CFI	TLI
AARC-gains in cognition	**−0.23 (−0.31; −0.16)**	**−0.06 (−0.09; −0.03)**	0.998	0.993
AARC-losses in cognition	**−0.37 (−0.46; −0.28)**	**−0.05 (−0.09; −0.02)**	0.997	0.992
IQCODE-self	**2.92 (2.58; 3.58)**	0.90 (0.76; 1.18)	0.996	0.987

All models are adjusted for age, sex, education, and working status. CFI, Comparative Fit Index; TLI, Tucker-Lewis Index. Bold text indicates that Confidence Intervals (CI) do not span 0.

### Subjective cognitive aging concepts as predictors of working memory over time

As we found the presence of multiple growth trajectories for WM in the sample, results of LGCM exploring average change in WM are presented in Supplementary Table S3. Here we describe results from the Three-Class GMM-CV for WM (Table 3). Class 1 comprised 182 participants (6% of the sample) with a mean WM score of 31.20 at baseline, and whose WM declined over the study period (mean slope = −2.48; 95% CI: −3.36; −1.60). Class 2 comprised 119 participants (4% of the sample) with a mean WM score of 23.12 at baseline, and whose WM score increased over the study period (mean slope = 3.28; 95% CI: 2.42; 4.15). Class 3 comprised 2,998 participants (90% of the sample) with a mean WM score of 30.11 at baseline, and whose score did not significantly change over time. Those with higher levels of AARC-gains in cognition (Odds Ratio = 1.08; 95% CI: 1.03; 1.14) and higher AARC-losses in cognition (Odds Ratio = 1.10; 95% CI: 1.04; 1.16) were more likely to be in Class 2 than Class 3. Scores on the IQCODE-self did not predict class membership.

**Table 3:** Three-class growth mixture modeling—class varying for working memory.

	Class 1 (*N* = 182; 6%)	Class 2 (*N* = 119; 4%)	Class 3 (*N* = 2,998; 90%)
	Estimate (95% CI)	Estimate (95% CI)	Estimate (95% CI)
Intercept	31.20 (29.69; 32.71)	23.12 (21.61; 24.63)	30.11 (28.73; 31.49)
Slope	**−2.48 (−3.36; −1.60)**	**3.28 (2.42; 4.15)**	−0.26 (−1.00; 0.47)
Variance–covariance			
Intercept	15.96 (10.93; 20.99)	2.82 (0.91; 4.75)	2.53 (1.92; 3.14)
Slope	3.64 (1.48; 5.80)	0.55 (−0.54; 1.63)	−0.84 (−1.13; −0.54)
Intercept-slope	1.05 (0.72; 1.37)	1.05 (0.72; 1.37)	1.05 (0.72; 1.37)
Predictors of class membership for working memory composite score using multinomial logistic regression models			
	Odds ratio (95% CI)	Odds ratio (95% CI)	Odds ratio (95% CI)
AARC-gains in cognition	1.05 (0.99; 1.10)	**1.08 (1.03; 1.14)**	Reference
AARC-losses in cognition	1.06 (1.00; 1.11)	**1.10 (1.04; 1.16)**	Reference
IQCODE-self	0.70 (0.21; 2.29)	1.92 (0.58; 6.33)	Reference

Models are adjusted for age, sex, education, and working status. Text in bold indicates statistical significance (i.e., estimates do not span 0; odds ratio does not span 1).

## Discussion

This study investigated for the first time whether, among cognitively healthy UK individuals aged 65+, trajectories of two-year change in VR and WM can be predicted by different aspects of subjective cognitive aging including participants’ baseline levels of AARC-gains and AARC-losses in cognition and SCD. VR remained stable over 2 years for all participants. Higher AARC-gains and higher AARC-losses in cognition at baseline were all associated with poorer VR at baseline and slightly greater decline in VR over time. Greater SCD at baseline was instead associated with better VR solely at baseline. Trajectories of WM instead varied among participants. A small group (Class 1, 6% of participants) scored high on WM at baseline but showed a decrease in WM over time. Another small group (Class 2, 4% of participants) scored low on WM at baseline but showed a constant improvement in WM over time and, at the end of the study, participants in this group scored higher on WM than those in Class 1. Most participants (Class 3, 90% of participants) scored almost as high as Group 1 in WM at baseline but, differently from those in Group 1, their WM remained stable over time. Those in Class 2 reported, at baseline, highest levels of AARC-gains and AARC-losses in cognition.

Overall, although associations were of small size, results suggest that, among cognitively healthy older adults, both higher AARC-gains and higher AARC-losses in cognition may indicate poorer concurrent VR and WM. The association between higher AARC-losses and poorer cognition is in line with our hypothesis and previous cross-sectional evidence. Indeed, poorer VR and WM were previously found cross-sectionally related to higher AARC-losses across life domains in samples of US and Norwegian older adults (37–39). Moreover, in US and Norwegian samples lower AARC-gains across life domains were also associated with higher performance on cognitive tasks assessing VR and WM (37, 38).

Previous evidence suggests that the cross-sectional association between higher AARC-losses in cognition and poorer objective cognition may be due to individuals with high AARC-losses having experienced greater decline in cognition over the past decades (40). The fact that higher AARC-gains have consistently been cross-sectionally associated with poorer objective cognition suggests the presence of a not yet well understood mechanism between AARC-gains and cognition. It may be that those individuals who have been experiencing an objective trajectory of cognitive decline, which is reflected in their higher levels of AARC-losses (40), try to compensate for age-related cognitive decline with engagement in cognitively stimulating activities which may lead to higher perceived gains. Indeed, participants reporting higher AARC-gains are typically more engaged in cognitively stimulating social and cultural activities (Sabatini et al., under review) and in adaptive behaviors (52). Moreover, greater engagement with social media was, for example, found to predict future increase in AARC-gains (53). Alternatively, these individuals may just be more aware of the aging process and the positive and negative changes it involves.

Moreover, although higher AARC-gains in cognition were reported by individuals with poorer WM at baseline, higher perceived age-related cognitive gains predicted future increase in WM among cognitively healthy older adults who initially scored low on WM tasks. It may be that, because individuals perceiving many age-related gains in cognition have internalized more positive age-related stereotypes (54), their positive attitude toward aging may facilitate future cognitive improvement. The observed improvement in WM among those individuals with poorer cognition at baseline may be due to practice effect (55), which is common among cognitively healthy individuals who repeat the same tasks at multiple assessment waves. As our sample comprised solely cognitively healthy individuals at baseline, whether higher AARC-gains in cognition are useful to predict future cognitive improvement and/or lower cognitive decline among individuals with pathological decline, including mild cognitive decline, is unknown.

In this study, AARC-losses emerged as a better indicator of cognition than SCD. Indeed, whereas higher AARC-losses in cognition were a useful indicator of poorer VR at the cross-sectional level, greater SCD was minimally related to better VR. Moreover, whereas higher AARC-losses were cross-sectionally related to poorer WM, SCD was not related to WM. The discrepancy in the results found for AARC and SCD likely reflects the features of the particular instrument used to operationalize SCD in this study. The IQCODE, developed for the screening and evaluation of dementia, queries participants not only about memory changes, but fairly significant functional changes (e.g., handling money for shopping, knowing how to use familiar machines around the house) and whose presence could indicate the presence of dementia. With the inclusion of only cognitive healthy older adults in the sample, few people would be expected to endorse this degree of a cognitive change, or the type of memory change queried by the IQCODE (remembering things about family and friends, remembering address and telephone number).

Other differences between the AARC and IQCODE may also have contributed to the current findings. First, whereas the AARC questionnaire provides separate scores for perceived cognitive improvement and perceived cognitive decline, the IQCODE-self positions individuals’ scores on a continuum from cognitive decline to cognitive improvement. As in this study we found that higher scores on both AARC-gains and AARC-losses were related to poorer cognition at cross-sectional level, it may be that the separate effects of perceived cognitive improvement and perceived cognitive decline cancel each other’s out in a measure (i.e., IQCODE-self) that converges perceptions of cognitive gains and losses in an overall score. Second, whereas in the IQCODE-self, individuals are asked to compare their current cognitive performance with how they performed 10 years ago, in the AARC questionnaire participants are purposefully not asked to compare their current cognitive functioning with a given timeframe. Third, the AARC questionnaire specifically asks participants to reflect on those changes they attribute to aging. Taken together, these differences across measures reinforce the importance of tailoring subjective cognitive instruments with regard to type of changes, time frame, and reference group (i.e., oneself, others one’s age) in order to best detect the specific type of cognitive change in question. Results of this study suggest that the AARC questionnaire is well suited to detect subtle, cross-sectional cognitive differences among cognitively healthy older adults.

While AARC-losses in cognition were associated with cognitive performance at baseline, they did not predict future cognitive decline. It may therefore be that, in cognitively healthy individuals, AARC-losses in cognition better capture greater past cognitive decline and poorer current cognitive functioning rather than being a predictor of future cognitive decline. In fact, as higher AARC-losses in cognition at baseline were reported by those whose WM performance was low at baseline but increased over time, it may be that higher AARC-losses in cognitively healthy older adults act as a motivating factor for engagement in cognitive activities that may help to regress from a state of age-related cognitive decline to intact cognition.

### Strengths and limitations

This study has several strengths. First, this is the first study exploring the predictive role of levels of AARC-gains and AARC-losses in cognition for future trajectories of cognitive change. Second, the large sample which made it possible to conduct sophisticated data analyses such as GMM is a strength. Third, different from previous studies which linked unidirectional measures of SPA (56) to cognitive change, we assessed the coexistence of positive and negative perceived age-related changes in cognition. Fourth, we used several criteria to exclude from the study sample individuals who may have had pathological cognitive decline. This increases the value of our results in the area of prevention of cognitive decline. Last, this is the first study to our knowledge to incorporate both AARC and SCD, related constructs which to date have been examined separately.

Nonetheless, this study has some limitations. First, the UK PROTECT study sample includes individuals with above average education and self-rated health. As these factors play a role in cognitive health maintenance (57), our results should be generalized with caution to the broader UK population aged 65+. Second, the sample was predominantly Caucasian and therefore is not representative of the broader population of older adults. Third, although a 2-year follow-up allowed us to detect change in WM in a subgroup of participants, longer follow-ups may be needed to detect change in VR. Fourth, measures of cognitive function were limited, and it may be that a more comprehensive neuropsychological assessment would reveal a stronger or perhaps different pattern of results. However, despite including only four cognitive tasks, the UK PROTECT cognitive test battery has been shown to be sensitive to detect cognitive changes in clinical trials (43). Fifth, it is unknown whether the observed objective changes in cognition, especially in the subgroup that experienced a significant decline in objective cognition, fall in the normal range or are indicative of cognitive impairment. Hence, even though at baseline all participants were free of objective cognitive impairment we cannot rule out the possibility that there may have been some individuals who developed cognitive impairment over the study period and impacted self-awareness at baseline.

## Conclusion

Consistent with previous evidence, the study results suggest that higher levels of perceived age-related cognitive changes, whether gains or losses, may be indicators of slightly poorer concurrent verbal reasoning and working memory. Nonetheless, this study also found, for the first time, that slightly higher perceived gains and losses in cognition were reported by a small group of individuals who showed a significant improvement in working memory ability over 2 years. Hence, among cognitively healthy individuals, both perceived gains and losses in cognition may act as motivational resources that facilitate improvement in working memory.

## Data availability statement

The data analyzed in this study is subject to the following licenses/restrictions: UK PROTECT data is available upon request and approval from the UK PROTECT steering committee. Requests to access these datasets should be directed to https://www.protectstudy.org.uk.

## Ethics statement

UK PROTECT obtained ethical approval from the London Bridge NHS Research Ethics Committee and Health Research Authority (Ref:13/LO/1578). The studies were conducted in accordance with the local legislation and institutional requirements. Written informed consent for participation was not required from the participants or the participants’ legal guardians/next of kin in accordance with the national legislation and institutional requirements.

## Author contributions

SS: Conceptualization, Data curation, Formal analysis, Funding acquisition, Writing – original draft. SCo: Writing – review & editing. SCh: Writing – review & editing. CB: Writing – review & editing. HB: Writing – review & editing. AC: Writing – review & editing. BS: Writing – review & editing.

## Footnotes

^1^ https://www.protectstudy.org.uk
