# Supplementary material for: Cognitive trajectories: exploring the predictive role of subjective cognitive decline and awareness of age-related changes for cognitive functioning
Source: Front Psychiatry. 2023 Oct 19;14:1270798. doi: 10.3389/fpsyt.2023.1270798 (PMC10620507; doi:10.3389/fpsyt.2023.1270798)
Supplement: Supplementary file 1 [file Data_Sheet_1.PDF]

Cognitive trajectories: exploring the predictive role of subjective cognitive aging for cognitive functioning

Supplementary table 1. Testing increasing number of classes in latent class growth analysis, growth mixture modeling - class invariant, and growth mixture modeling – class varying for verbal reasoning.

Fit statistics	1 Class	2 Class	3 Class	4 Class
LCGA				
LL	-31496.404	-30255.042	-29679.572	-29406.434
BIC	63098.121	60639.701	59513.065	58991.093
ssaBIC	63056.814	60588.862	59513.065	58921.189
Entropy	1.00	0.715	0.777	0.772
Adj LMR-LRT		2384.604	1101.814	530.998
(p)		0.0000	0.0000	0.0000
BLRT		-31496.404	-30253.148	-29682.858
(p)		0.0000	0.0000	0.0000
Group size (%)	3299 (100%)			
C1		1603 (49%)	1807 (55%)	1327 (40%)
C2		1694 (51%)	978 (30%)	1272 (39%)
C3			512 (16%)	363 (11%)
C4				336 (10%)
GMM-CI				
LL	-29208.682	-29195.811	-29185.044	-29179.601
BIC	58546.981	58545.543	58548.311	58561.730
ssaBIC	58496.142	58485.171	58478.407	58482.294
Entropy	1.00	0.982	0.964	0.843
Adj LMR-LRT		24.724	17.295	8.232
(p)		0.0468	0.5849	0.2841
BLRT		-29208.682	-29194.047	-29183.887
(p)		0.0000	0.0000	0.0806
Group size (%)	3299 (100%)			
C1		3281 (99%)	3249 (99%)	17 (1%)
C2		17 (1%)	32 (1%)	232 (7%)

C3			16 (0.5%)	22 (1%)
C4				3027 (92%)
GMM-CV				
LL	-29208.682	-29186.248	-29177.369	-29162.359
BIC	58546.981	58542.618	58565.367	58575.851
ssaBIC	58496.142	58475.891	58482.753	58477.350
Entropy	1.00	0.721	0.688	0.750
Adj LMR-LRT		43.787	17.275	26.910
(p)		0.0198	0.1931	0.0000
BLRT		-29208.682	-29186.220	-29176.146
(p)		0.1111	0.6667	1.0000
Group size (%)	3299 (100%)			
C1		3101 (94%)	2889 (88%)	30 (1%)
C2		197 (6%)	130 (4%)	247 (7%)
C3			279 (8%)	2893 (88%)
C4				128 (4%)

Notes: All models are adjusted for age, sex, education, and working status. LCGA= Latent class growth analysis. GMM-CI= Growth mixture modeling - class invariant. GMM-CV – Growth mixture modeling – class varying. In the LCGA, variances and covariances are set to zero. In a GMM-CI, variances and covariances are freed between classes but not within classes (variances fixed across classes). In a GMM-CV, all parameters are freed. LL= Log-likelihood. BIC= Bayesian Information Criterion. ssaBIC= Sample size adjusted BIC. Adj LMR-LRT= Lo-Mendell-Rubin adjusted Likelihood Ratio Test. BLRT= Bootstrap Likelihood Ratio Test. C1-C4 = Class 1-Class 4.

Supplementary Table 2. Testing increasing number of classes in latent class growth analysis, growth mixture modeling - class invariant, and growth mixture modeling – class varying for working memory.

Fit statistics	1 Class	2 Class	3 Class	4 Class
LCGA				
LL	-21560.267	-21000.711	-20717.848	-20586.314
BIC	43225.852	42131.045	41589.621	41350.858
ssaBIC	43184.545	42080.206	41529.250	41280.954
Entropy	1.00	0.552	0.709	0.707
Adj LMR-LRT		1074.885	-20998.012	239.894
(p)		0.0000	0.0143	0.0251
BLRT		-21560.267	-20998.012	-20711.196
(p)		0.0000	0.0000	0.0000
Group size (%)				
C1	3299 (100%)	1671 (51%)	280 (8%)	1492 (45%)
C2		1628 (49%)	2242 (68%)	142 (4%)
C3			777 (24%)	157 (5%)
C4				1508 (46%)
GMM-CI				
LL	-20592.874	-20463.244	-20354.324	-20315.796
BIC	41315.370	-20463.244	40886.878	40834.127
ssaBIC	41264.531	41020.043	40816.974	40754.691
Entropy		0.915	0.916	0.930
Adj LMR-LRT		249.014	203.552	82.930
(p)		0.0000	0.0000	0.0001
BLRT		-20592.874	-20460.288	-20358.967
(p)		0.0000	0.0000	0.0000
Group size (%)				
C1	3299 (100%)	115 (3%)	115 (3%)	3075 (93%)
C2		3184 (97%)	91 (3%)	103 (3%)
C3			3093 (94%)	119 (4%)
C4				2 (0.1%)
GMM-CV				
LL	-20592.874	-20368.057	-20281.213	-20260.575
BIC	41315.370	40906.242	40773.062	40772.293
ssaBIC	41264.531	40839.516	40690.448	40673.792
Entropy		0.619	0.769	0.761
Adj LMR-LRT		438.802	152.578	14.445
(p)		0.0000	0.0001	0.0460
BLRT		-20592.874	-20359.385	-20267.976
(p)		0.0000	0.0000	0.3077
Group size (%)				
C1	3299 (100%)	268 (8%)	182 (6%)	6 (0.2%)
C2		3031 (92%)	119 (4%)	2943 (89%)
C3			2998 (90%)	128 (4%)
C4				222 (7%)

Notes: All models are adjusted for age, sex, education, and working status. LCGA= Latent class growth analysis. GMM-CI= Growth mixture modeling - class invariant. GMM-CV –

Growth mixture modeling – class varying. In the LCGA, variances and covariances are set to zero. In a GMM-CI, variances and covariances are freed between classes but not within classes (variances fixed across classes). In a GMM-CV, all parameters are freed. LL= Log-likelihood. BIC= Bayesian Information Criterion. ssaBIC= Sample size adjusted BIC. Adj LMR-LRT= Lo-Mendell-Rubin adjusted Likelihood Ratio Test. BLRT= Bootstrap Likelihood Ratio Test. C1-C4 = Class 1-Class 4.

Supplementary table 3. Latent growth curve model estimating baseline levels and change over time in working memory.

**Working memory**				
	Intercept mean	Slope mean	CFI	TLI
Unconditional model	32.20 (31.91; 32.77)	**1.10 (0.94; 1.41)**	0.947	0.843
Variance	5.89 (5.77; 6.12)	0.41 (0.35; 0.53)		
Latent growth curve model estimating predictors of working memory at baseline and over time				
Predictors	Intercept mean	Slope mean	CFI	TLI
AARC-gains in cognitive functioning	**-0.03 (-0.04; -0.02)**	**0.004 (0.002; 0.01)**	0.949	0.847
AARC-losses in cognitive functioning	**-0.07 (-0.07; -0.06)**	**0.02 (0.02; 0.03)**	0.949	0.846
IQCODE-self	**0.30 (0.29; 0.32)**	**0.04 (0.03; 0.05)**	0.948	0.845

Notes: All models are adjusted for age, sex, education, and working status.
CFI= Comparative Fit Index. TLI= Tucker-Lewis Index.
Bold text indicates that Confidence Intervals (CI) do not span 0.
